# Transoral Thyroidectomy: Initial Results of the European TOETVA Study Group

**DOI:** 10.1007/s00268-023-06932-7

**Published:** 2023-02-17

**Authors:** Melisa Arikan, Philipp Riss, the European TOETVA Study Group, Christian Scheuba, Stefan Schopf, Elias Karakas, Günther Klein, Linda Michlmayr, Martin Schardey, Peter Busch, Michael Hermann, Thomas Grabner, Özer Makay, Murat Özdemir, Ziya Karimov, Gianlorenzo Dionigi, Andrea Casaril

**Affiliations:** Division of Visceral Surgery, Department of General Surgery, Medical University of Vienna, Waehringer Guertel 18-20, 1090 Vienna, Austria

## Abstract

**Background:**

The aim of this study was to evaluate a new surgical technique by the European Transoral Endoscopic Thyroidectomy Vestibular Approach (TOETVA) Study Group.

**Methods:**

This study included 391 patients (47 [(12%]) male and 344 [(88%)] female) who had undergone endoscopic thyroid or parathyroid surgery via the vestibular approach between February 2016 and May 2022 at nine centers. The data were analyzed with regard to complications, surgery time and specimen retrieval.

**Results:**

Overall, 376 (96.2%) TOETVA and 15 (3.8%) transoral endoscopic parathyroidectomy vestibular approach interventions were performed with an average surgery time of 145 (± 61.2) minutes and 509 nerves at risk. The specimens were retrieved via a transoral vestibular and retroauricular approach in 66 (16.9%) patients and via a transaxillary approach in 8 (2%). Benign histology including Grave’s disease was identified in 272 (69.6%) patients, 1 (0.3%) presented noninvasive follicular thyroid neoplasms with papillary-like nuclear features, and 103 (26.3%) showed differentiated thyroid carcinoma. Solitary parathyroid adenoma were removed in 15 (3.8%) patients. Conversion to open surgery was necessary in 13 (3.3%) and revision had to be performed in 2 (0.5%) patients. Transient recurrent laryngeal nerve palsy (RLNP) was present in 18 (4.6%) and permanent RLNP in 2 (0.5%) patients. Fifteen (3.8%) patients experienced transient hypoparathyroidism after thyroidectomy. No case of permanent hypoparathyroidism was observed. Postoperative surgical site infection occurred in 1 (0.3%) patient. Despite a higher rate of sensory and motor disorders and skin discoloration at discharge, permanent disorders were present in only 3 (0.8%) and 16 (4.1%) patients, respectively.

**Conclusion:**

Our results show that transoral endoscopic surgery, performed by experienced endocrine surgeons, is a safe alternative to conventional thyroid surgery.

## Introduction

Since the 1880s, the Kocher cervical incision has been used as surgical access in thyroid and parathyroid surgery. To reduce the size of the scar, the surgical technique has been modified several times over the years, resulting in the development of minimally invasive open thyroid surgery with an incision of 3–5 cm [1–3]. Nevertheless, great interest developed in a surgical technique that did not yield a visible scar. Anuwong et al. [4] described the transoral endoscopic thyroidectomy vestibular approach (TOETVA) in a case series of 60 patients. Over the past six years, transoral thyroid surgery has become widely popular worldwide. Currently, only large case series from Asia have so far been published and data from single European centers are scarce. Thus, no significant statement on transoral thyroid surgery can be made regarding European patients who not only differ from Asian patients in BMI and physiognomy but also originate/live in a widely endemic goiter region [4, 5]. To overcome this deficit, endocrine surgeons from different institutions have come together to form the European TOETVA Study Group to evaluate and further improve transoral surgery in Europe. The aim of this study was to present the initial analysis of the data emerging from this research endeavor.

## Material and methods

The European TOETVA Study Group comprises nine institutions in Austria, Germany, Italy and Turkey. All patients who had undergone endoscopic thyroid or parathyroid surgery via vestibular approach from February 2016 to May 2022 at those centers were included in this study. Initial cases of patients undergoing TOETVA in each institution were included in this study. The selection of patients differed slightly from center to center; similar perioperative outcomes were identified among the patients.

Data were collected by each institution. Furthermore, for clinics using the EUROCRINE platform, a special TOETVA module was provided for data entry and export. After approval from each institution, the data were pooled and analyzed retrospectively.

The demographic data include age and sex (male, female). Intraoperative data were analyzed regarding surgery duration (in minutes), procedure applied, type of surgery (TOETVA, transoral endoscopic parathyroidectomy vestibular approach [TOEPVA], combined procedure), specimen retrieval (vestibular, retroauricular, axillary), histology, intraoperative complications, conversion (defined as intraoperative change to unplanned open surgery), and revision (defined as reoperation due to complication). The reasons for conversion and/or revision were documented.

The postoperative data include such complications as transient and permanent recurrent laryngeal nerve palsy (RLNP) and hypoparathyroidism, infection, bleeding, seroma, scars, skin discoloration, sensory disorders, motor disorders and duration of hospital stay.

Both postoperative hypoparathyroidism and RLNP, respectively, with no recovery for over 6 months were defined as permanent. Intermittent neuromonitoring was used in all patients.

TOETVA-associated complications were divided into the following groups: perioral, mandibular margin and neck. Motor disorder caused by injury to the mental nerve was categorized as hanging corner of the mouth (unilateral or bilateral) or limited motor function of the neck.

### Statistical analysis

Data analysis was performed using SPSS® version 23.0 (SPSS, Chicago, Illinois, USA). Data with a significance level set to *p* < 0.05 were considered statistically significant. Metric parameters were described by mean values, the corresponding standard deviation, minimum and maximum. Ordinal and nominal parameters were described by absolute and relative numbers.

## Results

### Demographic data

Overall, 391 patients, 344 (88%) female and 47 (12%) male, with 509 nerves at risk and a mean age of 40.8 ± 13.4 (15–77) years as shown in Table 1, were included in the analysis. The average hospital stay was 2.5 ± 1.2 (1–6) days. The numbers and time periods of performed TOETVAs in each institution are shown in Table 2*.*

**Table 1:** Demographic data, hospital stay and nerves at risk of patients

	Mean ± SD (range)		*n* (%)
Age	40.8 ± 13.4 (15–77) years	Sex	
		Female	344 (88)
		Male	47 (12)
Hospital stay	2.5 ± 1.2 (1–6) days	Nerves at risk	509

SD: Standard deviation

**Table 2:** Number of performed transoral endoscopic surgeries and their time periods in each institution

Institution	*n*	Time period
University of Milan, Milan, Italy	82	2016–2021
Pederzoli Hospital, Peschiera del Garda, Verona, Italy	63	2017–2022
Medical University of Vienna, Vienna, Austria	53	2018–2022
Maria Hilf Hospital, Alexianer GmbH, Krefeld, Germany	47	2017–2022
Landesklinikum Wiener Neustadt, Wiener Neustadt, Austria	40	2017–2020
RoMed Hospital, Bad Aibling, Germany	39	2020–2021
Ege University Hospital, Izmir, Turkey	34	2018–2020
Agatharied Hospital, Hausham, Germany	19	2019–2021
Klinik Landstraße, Vienna, Austria	14	2020–2022

### Surgery (and intraoperative complications)

TOETVA was performed in 376 (96.2%) patients and TOEPVA in 15 (3.8%) patients. In 6 (1.5%) cases, TOEPVA was combined with thyroid surgery including 5 hemithyroidectomies and one total thyroidectomy.

Total thyroidectomy was performed in 117 (29.9%) patients, subtotal thyroidectomy or Dunhill surgery in 9 (2.3%), hemithyroidectomy in 245 (62.7%), resection of the isthmus or median cyst in 11 (2.8%) and parathyroidectomy in 15 (3.8%) patients.

Specimen retrieval was vestibular in 317 (81.1%), retroauricular in 66 (16.9%) and axillary in 8 (2%) patients. The mean operative time was 145 ± 61.2 (29–420) minutes.

The average surgery time was 174 ± 70.7 (45–420) minutes for total and subtotal thyroid surgery, 135.3 ± 52 (29–370) for lobectomies, 113 ± 47.5 (65–216) for isthmus and median cyst resection and 115.4 ± 45.2 (45–180) minutes for parathyroidectomies. On average, combined surgeries of the parathyroid and thyroid gland lasted 112 ± 32.7 (70–160) minutes.

Revision was necessary in 2 (0.5%) patients due to bleeding. Conversion was performed in 13 (3.3%) patients. In 6 (1.5%) patients, bleeding was the reason for conversion. Conversion was performed in 2 (0.5%) patients due to adiposity, combined with short neck and thyroid specimens of more than 40 ml volume, and in single cases (0.3%) due to tracheal tear, carcinoma, skin injury (punctual perforation of the skin), endobag rupture or CO_2_ embolism. Further, subcutaneous tumor seeding occurred in 1 (0.3%) case. Minor skin burn injury (without conversion) was seen in 2 (0.5%) patients. Main intraoperative data are depicted in Table 3. Transient RLNP was present in 18 (4.6%) and permanent RLNP in 2 (0.5%) patients. Transient hypoparathyroidism occurred in 15 (3.8%) patients. No patient experienced permanent hypoparathyroidism.

**Table 3:** Intraoperative data

Surgery time (mean ± SD, range)	145 ± 61.2 (29–420) minutes
	*n* (%)
*Type of surgery*	
Hemithyroidectomy	245 (61.7)
Total thyroidectomy	117 (29.5)
Subtotal thyroidectomy	1 (0.3)
Isthmus resection	8 (2)
Median cyst resection	3 (0.8)
Dunhill surgery	8 (2)
Parathyroidectomy	15 (3.8)
*Specimen retrieval*	
Vestibular	322 (81.3)
Retroauricular	66 (16.7)
Axillary	8 (2)
Revision	2 (0.5)
Conversion	13 (3.3)
Other intraoperative complications	2 (0.5)
*RLNP*	
Transient	18 (4.7)
Permanent	2 (0.5)
*Hypoparathyroidism*	
Transient	15 (3.9)
Permanent	0 (0)

SD: Standard deviation, RLNP: recurrent laryngeal nerve palsy

### Histology

As seen in Table 4, among the patients, 272 (69.6%) presented with benign histology with thyroid surgery, including 21 (5.4%) patients with Grave´s disease and 1 (0.3%) patient with noninvasive follicular thyroid neoplasm with papillary-like nuclear features. Differentiated thyroid carcinoma developed in 103 (26.3%) individuals with 89 (22.8%) papillary thyroid carcinoma, 13 (3.3%) microcarcinoma and one (0.3%) follicular carcinoma. Fifteen (3.8%) adenoma of the parathyroid glands were identified.

**Table 4:** Patient histology

	*n* (%)
Benign	272 (69.6)
Grave’s disease	21 (5.4)
NIFTP	1 (0.3)
Carcinoma	103 (26.4)
Papillary thyroid carcinoma	89 (22.8)
Microcarcinoma	13 (3.3)
Follicular carcinoma	1 (0.3)
Adenoma of the parathyroid gland	15 (3.8)

NIFTP: Noninvasive follicular thyroid neoplasms with papillary-like nuclear features

### Postoperative complications

Sensory (perioral, mandibular margin, neck) and motor disorders were transient in 67 (17.1%) and 16 (4.1%) patients, respectively. In 1 (0.3%) patient, sensory disorder was temporarily described in the lobe of the ear. Among those with motor disorders, unilateral hanging of the mouth was transient in 4 (1%). In another case (0.3%), the transient motor disorder was prominent in the neck. Permanent sensory and motor disorders were present in 3 (0.8%) patients and in 1 (0.3%), respectively.

Skin discoloration, pain, swelling and seroma were transient in 49 (12.5%), 20 (5.1%) and 25 (6.4%) cases and in 1 (0.3%) case, respectively. Postoperative infection was present in 1 (0.3%) patient.

Other temporary postoperative complications, as depicted in Table 5, were skin lesion in 3 (0.8%) and induration of the chin in 2 (0.5%) patients. Dysphonia and chewing problems were temporarily present in 1 (0.3%) patient each. In 1 (0.3%) patient, skin lesion in terms of a scar after intraoperative burning was permanent.

**Table 5:** TOETVA-/TOEPVA-associated complications by their localizations

	*n* (%)			
	Total	Perioral	Mandibular margin	Neck
Sensory disorder	67 (17.3)	29 (7.5)	31 (8)	4 (1.1)*
Motor disorder	16 (3.6)	4 (1)	0 (0)	1 (0.3)
Pain	20 (5.2)	3 (0.8)	3 (0.8)	2 (0.5)
Swelling	25 (6.5)	16 (4.1)	3 (0.8)	2 (0.5)
Skin discoloration	49 (12.7)	1 (0.3)	11 (2.8)	0 (0)

TOETVA: Transoral endoscopic thyroidectomy vestibular approach, TOEPVA: transoral endoscopic parathyroidectomy vestibular approach

*Earlobe in one patient

## Discussion

Over the years, there was a keen interest in developing a procedure in thyroid surgery, which would leave no visible scar.

Miccoli et al. were able to develop the first minimally invasive video-assisted thyroidectomy which has become a globally applied technique [6–9]. Witzel et al. [10] reported a transoral thyroid resection with a sublingual approach via the oral cavity, which was first performed on cadavers. Karakas et al. [11] published preliminary results in 2011, but this technique was discontinued due to complications.

Anuwong et al. [4] were the first to report on TOETVA in a case series of 60 patients with a low rate of complications. Currently, large case series of TOETVA have been published in Asia [12].

In our pooled data analysis, we focused our attention on complications associated with TOETVA/TOEPVA and specimen retrieval. Our study presents the largest multicenter analysis of this topic in Europe.

Table 2 shows the numbers of performed cases in each participating institution. The first TOETVAs were performed in 2016.

Mean operation time was 145 ± 61.2 min, as shown in Table 3. The endpoint of the procedure was defined differently in the institutions. In 14 patients, the end of surgery was documented when receiving the intraoperative result of frozen section (instead of skin-to-skin time). Nevertheless, the data are comparable to those in the literature demonstrating operative time to vary between 60 and 365 min, which, however, is longer than in open surgery. This surgery duration has been explained to be due to the learning curve and extension of the indication in some centers that performed a combination of techniques to operate on thyroid specimens >50 ml in volume [5].

Hospital stay (Table 1) varied from one to six days. The difference in discharge refers to the difference in country-specific hospital policies. In a systematic review, the average hospital stay was seen to be comparable with one to eight days [13].

### Specimen retrieval

For the retrieval of larger specimens, TOETVA was combined with endoscopic cephalic-access thyroid surgery using the retroauricular approach, which was first performed in 2006 and is still considered a safe and feasible procedure with excellent cosmetic results [14, 15]. Combining this technique with TOETVA, a 12-mm trocar was inserted retroauricularly. The first thyroid lobectomy for large specimens without a visible scar via a transoral vestibular and retroauricular approach (TOVARA) was performed in 2017 and can be applied to thyroid glands with a volume of more than 40 ml [16, 17]. As shown in Table 3, retroauricular specimen retrieval was performed in 66 (16.9%) patients. In one patient (0.3%), transient sensory disorder in the earlobe was documented. The single-port axillary approach with a 12-mm trocar was used in 8 (2%) patients, as shown in Table 3. The axillary approach in our study did not involve any surgical steps or type of dissection of the bilateral axillo-breast approach [18, 19]. The combination of the axillary approach with TOETVA was used by one surgeon from the European TOETVA Study Group to reduce trauma on the lips during specimen retrieval. In 317 (81.1%) patients, the specimen was retrieved via vestibular approach. Postoperative complications, such as location of sensory and motor disorders, differed according to approach.

### Complications and conversion

As shown in Table 3, postoperative temporary hypoparathyroidism developed in 15 (3.8%) patients. No permanent hypoparathyroidism was detected. Due to the optimum view of video endoscopy and the direction of surgery from the upper pole downwards, upper parathyroid glands can almost always be seen and preserved. Another explanation for our results could be a higher concentration during surgery to avoid complications while implementing the new procedure. Recently published outcomes regarding transient and permanent hypoparathyroidism after transoral thyroidectomy range between 1 and 18.2% and between 0 and 2%, respectively [20–23]. Similar results have been described in open thyroid surgery, with rates of transient and permanent hypoparathyroidism of up to 33.6% and 8%, respectively [24–27]. Considering our data, transoral thyroid surgery yielded improved results regarding permanent hypoparathyroidism.

Transient RLNP was present in 18 (4.6%) patients, and 2 (0.5%) showed permanent RLNP. In one patient who showed initially postoperative bilateral RLNP, an improvement was seen after six months with regression of the palsy on one side. Currently, this patient suffers from permanent unilateral RLNP and is receiving vocal training. Other TOETVA case series have produced similar results of transient and permanent RLNP of 1.3–7.6% and 0–2%, respectively [4, 13, 21, 22], which is also comparable to conventional surgery [27, 28].

Therefore, regarding the most common adverse events in thyroid surgery (RLNP and hypoparathyroidism), TOETVA and the conventional technique are to be considered comparable.

Conversion had to be performed in 13 patients, as shown in Table 3. The most common reason for conversion was bleeding (six patients), which was mainly caused by injury of the anterior jugular vein. In 2 (0.5%) obese patients, conversion was already necessary at the beginning of surgery. In one of those patients (0.3%), conversion was necessary directly at the beginning of surgery because the subplatysmal space was not reached due to an improper/unsuitable angle between the chin and the thyroid region. In this patient, extension of the neck was not sufficiently feasible. Additionally, in another young obese patient with a short neck, thyroid volume was too high (>60 ml) to safely mobilize the specimen to reach the para- and retrothyroidal region. In contrast to our data, some studies have demonstrated that a higher BMI in patients undergoing TOETVA did not lead to a significant difference in cumulative complications [23, 29].

During dissection and tunneling between chin and neck, an unintentional skin perforation occurred in 1 (0.3%) patient and was immediately sutured. However, the positioning of the central trocar led to a worsening of the skin lesion and widening of the wound. Consequently, the team decided to convert to open surgery.

Similar to various multicenter studies describing conversion due to uncontrolled bleeding, our results yielded 6 (1.5%) conversions due to bleeding. In the current literature, another reason for conversion has been nodule size [21, 22]. Still, conversion can be avoided in patients with large specimens when alternative (i.e., retroauricular/axillary) approaches for specimen retrieval are used.

In one of our first cases, conversion was performed because of an intraoperative malignant frozen section result to obtain adequate lymphadenectomy. In one (0.3%) case, conversion was carried out due to endobag rupture.

In yet another patient (0.3%), tracheal injury was the reason for conversion. In this case, extensive thyroiditis was present. The thyroid tissue was firm and traction resulted in tracheal injury. Weng et al. reviewed the literature and found ten articles on tracheal injury following endoscopic thyroidectomy. In those cases, both intraoperative and delayed tracheal injury were reported [30]. Intraoperative tracheal perforation was caused especially by the Veress needle and accidental dissection, whereas thermal injury was more likely to cause delayed rupture resulting in tracheal necrosis [22, 31].

One patient (0.3%) with papillary thyroid carcinoma, subcutaneous nodules and central compartment recurrence was identified during one and a half years after TOETVA. Intraoperatively, there was no tumor and/or endobag rupture. Therefore, the only explanation for this subcutaneous tumor seeding is tumor manipulation. Nevertheless, transoral thyroid surgery should not only be considered for benign disease. Although rarely, such situations may also occur in open surgery. To prevent cancer seeding and local recurrence, Anuwong et al. routinely used a specimen pouch to enclose the specimen before extraction [12]. This procedure during specimen retrieval was also performed in all patients participating in the present study.

In 1 (0.3%) case, CO_2_ embolism occurred due to an anterior jugular vein damage with an energy-based device tip during air pocket preparation. The surgeon immediately attempted to seal the vein, but end-tidal CO_2_ rapidly increased. Conversion was performed on the basis of the anesthetist’s request.

As possible in any endoscopic operation, Anuwong et al. described subcutaneous emphysema in 3.5% of their patients with complete resolution within 24 h. No other complications such as tracheal or esophageal injury were encountered [20]. Nevertheless, CO_2_ embolism caused by an anterior jugular vein damage has been documented in the recent literature [31–33]. To prevent this complication, Hong et al. proposed to compress the external neck for a few minutes before insufflating CO_2_ for hemostasis of an unintentionally lacerated vein. Thus, CO_2_ entering the lacerated vein can be blocked [31].

Infection was a major concern in TOETVA due to possible contamination of wounds with bacteria of the oral cavity. Dionigi et al. [34] described 2% of surgical site infections in a prospective analysis which was carried out on more than 200 patients undergoing conventional open cervical exploration for thyroid disease. Our results showed infection after TOETVA in one patient (0.3%), even though intraoperative single-shot antibiosis was given. Two days after discharge, the patient had to return for treatment with parenteral antibiotics. No revision was necessary. According to the literature, surgical site infection after transoral thyroidectomy is a very rare complication [4, 13, 21, 35–38].

Revision was performed in 2 (0.5%) patients due to bleeding. In one (0.3%) of those patients, revision was performed under the same anesthesia. In contrast to open thyroidectomy, in which postoperative bleeding occurs in up to 1–2% [39], our results showed no higher risk of revision due to bleeding. In both cases, revision was performed via vestibular approach. Anuwong et al. reported postoperative hematoma in one patient (0.5%), which was treated with open surgery [20].

As shown in Table 5, the most common postoperative complications were sensory and motor disorders due to mental nerve injury, which were mainly transient. Minimal sensory disorders were permanent in 3 (0.8%) patients, and a motor disorder was likewise permanent in 1 (0.3%) patient, yet showed an improvement over time. Pain, swelling, skin discoloration and seroma were transient in all patients. Due to different follow-up standards in the participating institutions, the exact time of resolution of each complication cannot be defined in this analysis. Apart from the most frequently occurring postoperative complaints, one patient (0.3%) presented dysphonia at discharge which was transient due to laryngeal edema. Patients who suffered intraoperative burn injury of the skin all showed improvement of the scar. Subsequent to intraoperative burn, minimal skin lesion in terms of a scar was still visible in the last follow-up in 1 (0.3%) patient. Anuwong et al. described transient mental nerve injury in 3 (0.7%) patients [12]. Compared to the data published by those authors, a slightly higher rate of complications was observed in our patient population. This higher complication rate can be attributed to differences in physiognomy and BMI in such an endemic goiter area. In summary, the type and number of complications associated with TOETVA in this group of patients are comparable to published data [21, 22].

### Limitations

Due to the retrospective character of this register study, several limitations are to be reported. First, our investigation was based on a female-dominated study population, which results in the inability to analyze gender-specific differences. Further, due to heterogeneity, no general inclusion and exclusion criteria for TOETVA can be defined in this register study. As data collection was center-based, no statement can be made on the volume and learning curve of a single surgeon. Follow-up was performed at different times in the clinics, thus eliminating the possibility to elaborate on the exact resolution time of complications.

## Conclusion

Although TOETVA is a novel surgical technique and still in a process of continuous development, it represents a feasible alternative in thyroid surgery with low complication rates. The current advantage is limited to the excellent cosmetic outcome. Further prospective trials with pooled data are needed to answer scientific questions and to analyze the specific aspects of this procedure.

## Appendix

Parameters used in the first data collection (via EUROCRINE module or excel sheet) and analyzed in this study.

**Table:**

Surgery date	Day/month/year
Age	Years
Sex	Male
	Female
Hospital stay	Days
Surgery duration	In minutes
Specimen retrieval	Vestibular
	Axillary
	Retroauricular
Surgery performed	Total thyroidectomy
	Hemithyroidectomy
	Subtotal thyroidectomy
	Dunhill surgery
	Isthmus resection
	Median cyst resection
	Parathyroidectomy
Histology	Benign nodular goiter
	Grave´s disease
	NIFTP
	Papillary thyroid carcinoma
	Microcarcinoma
	Follicular carcinoma
	Adenoma of parathyroid gland
Postoperative hypoparathyroidism	Transient
	Permanent
Postoperative RLNP	Transient
	Permanent
Conversion	Yes (including reason)
	No
Revision	Yes (including reason)
	No
*Sensory disorder*	
Pain Swelling Skin discoloration	Perioral Mandibular margin Neck
Motor disorder	Hanging corner of the mouth (unilateral vs. bilateral) Neck

NIFTP: noninvasive follicular thyroid neoplasms with papillary-like nuclear features, RLNP: recurrent laryngeal nerve palsy
